# Transmembrane transport and stress response genes play an important role in adaptation of *Arabidopsis halleri* to metalliferous soils

**DOI:** 10.1038/s41598-018-33938-2

**Published:** 2018-10-31

**Authors:** Christian Sailer, Alicja Babst-Kostecka, Martin C. Fischer, Stefan Zoller, Alex Widmer, Pierre Vollenweider, Felix Gugerli, Christian Rellstab

**Affiliations:** 1 WSL Swiss Federal Research Institute, Birmensdorf, 8903 Switzerland; 2 Present Address: ETH Zürich, Institute of Integrative Biology, Zürich, 8092 Switzerland; 3 W. Szafer Institute of Botany, Polish Academy of Sciences, Krakow, 31512 Poland; 4 ETH Zürich, Institute of Integrative Biology, Zürich, 8092 Switzerland; 5 ETH Zürich, Genetic Diversity Centre, Zürich, 8092 Switzerland

## Abstract

When plants adapt to local environments, strong signatures of selection are expected in the genome, particularly in high-stress environments such as trace metal element enriched (metalliferous) soils. Using *Arabidopsis halleri*, a model species for metal homeostasis and adaptation to extreme environments, we identifid genes, gene variants, and pathways that are associated with soil properties and may thus contribute to adaptation to high concentrations of trace metal elements. We analysed whole-genome Pool-seq data from two metallicolous (from metalliferous soils) and two non-metallicolous populations (in total 119 individuals) and associated allele frequencies of the identified single-nucleotide polymorphisms (SNPs) with soil variables measured on site. Additionally, we accounted for polygenic adaptation by searching for gene pathways showing enrichment of signatures of selection. Out of >2.5 million SNPs, we identified 57 SNPs in 19 genes that were significantly associated with soil variables and are members of three enriched pathways. At least three of these candidate genes and pathways are involved in transmembrane transport and/or associated with responses to various stresses such as oxidative stress. We conclude that both allocation and detoxification processes play a crucial role in *A. halleri* for coping with these unfavourable conditions.

## Introduction

Local adaptation is a key evolutionary process allowing plants to cope with environmental changes and/or to colonize new and selective habitats. It is driven by natural selection acting on genetically controlled fitness traits^1^ and has received increasing attention in the last decade. As selection leaves distinct signatures in the genome, various landscape and population genomic approaches have been developed to (i) identify the regions in the genome that are putatively involved in local adaptation, (ii) find the environmental factors driving this process, but also (iii) pinpoint constraints decreasing local adaptation and convolute its detection^2,3^. When populations exhibit high levels of gene flow, strong selection pressure is required to maintain local adaptation. Hence, the interplay between gene flow and selection strength determines the genetic structure of populations as well as the possibility of detecting the genomic signature of adaptation^3^ and understanding its genomic basis.

Metalliferous (M) habitats exert a strong selection pressure on plant communities from high and potentially toxic concentrations of some trace metal elements (TMEs) in soils (*Thlaspi caerulescens*^4^, *Biscutella laevigata*^5^). Such high concentrations of TMEs can occur naturally, for example in rare serpentine soils^6^, or can result from anthropogenic activities (e.g. mining). Their toxic effect on growth, biochemistry, and physiology strongly depends on the bioavailability of these elements in the soil and on plant tolerance mechanisms^7^. At the cell level, TME stress can result from e.g. enzymatic dysfunction via TME binding to a functional domain and/or increased oxidative stress (OS^8^). Such reactions are amplified through the accumulation of some TMEs (e.g. zinc, Zn) within OS-prone organelles such as chloroplasts, causing injury to thylakoids and photosystems, consequently impairing photosynthesis^9,10^.

Populations of several plant taxa have locally adapted to harsh M environments^11^. While avoidance by exclusion is the most common mechanism of plant adaptation to TME toxicity, tolerance to metal stress relies on allocation and detoxification strategies at organ, tissue and cell level^8^. Increased TME tolerance can be achieved through allocation to e.g. older foliage organs^12^ and/or peripheral and physiologically less active tissues in the leaf vein^13^, leaf blade^14^, and within cells the vacuole^15^ or cell wall^16^.

In certain hypertolerant species, metal accumulation can exceed the concentrations found in non-hypertolerant species growing on non-metalliferous (NM) soils by several orders of magnitude. These hyperaccumulator species^17^ show exceptionally high transcription levels of many genes involved in metal transport, chelation, and sequestration^18^. Hyperaccumulators thus have a high potential for phyto-remediation of heavy metal contaminated soils or phyto-fortification of certain minerals for increased nutrition value. However, for efficient application of these approaches, the genetic basis of metal adaptation remains insufficiently understood^19^.

Phenotypic studies on adaptation to calamine or serpentine soils using species that thrive on both M and NM soils, so called pseudometallophytes, have revealed considerable intraspecific variation in metal tolerance and hyperaccumulation^5,9,20^. At the species level, this large quantitative variation is commonly associated with different edaphic origins of populations. In general, populations on NM soils accumulate lower quantities of metals than populations on M soils. Yet, under experimentally controlled conditions, when all plants are exposed to the same elevated metal concentrations, non-metallicolous populations often accumulate higher amounts of TME compared to metallicolous populations, thus also reaching the threshold concentration for hyperaccumulation^20–22^. Genome scans^11,23^ and quantitative genetic studies^24,25^ have shown the involvement of genomic regions and genes that underlie processes of internal metal transport, homeostasis and/or detoxification in leaves of hyperaccumulating plants. Still, these studies are based on genomic information and categorical assignment (e.g. M or NM soils) only and lack associations with quantitative environmental variables that characterize e.g. soil metal content. Furthermore, the existing analyses and interpretations are often gene-focused and thus offer only limited insight into the genetic basis of adaptation to environmental stress, despite increasing evidence suggesting that such adaptation is polygenic^26^.

**Figure 1:**
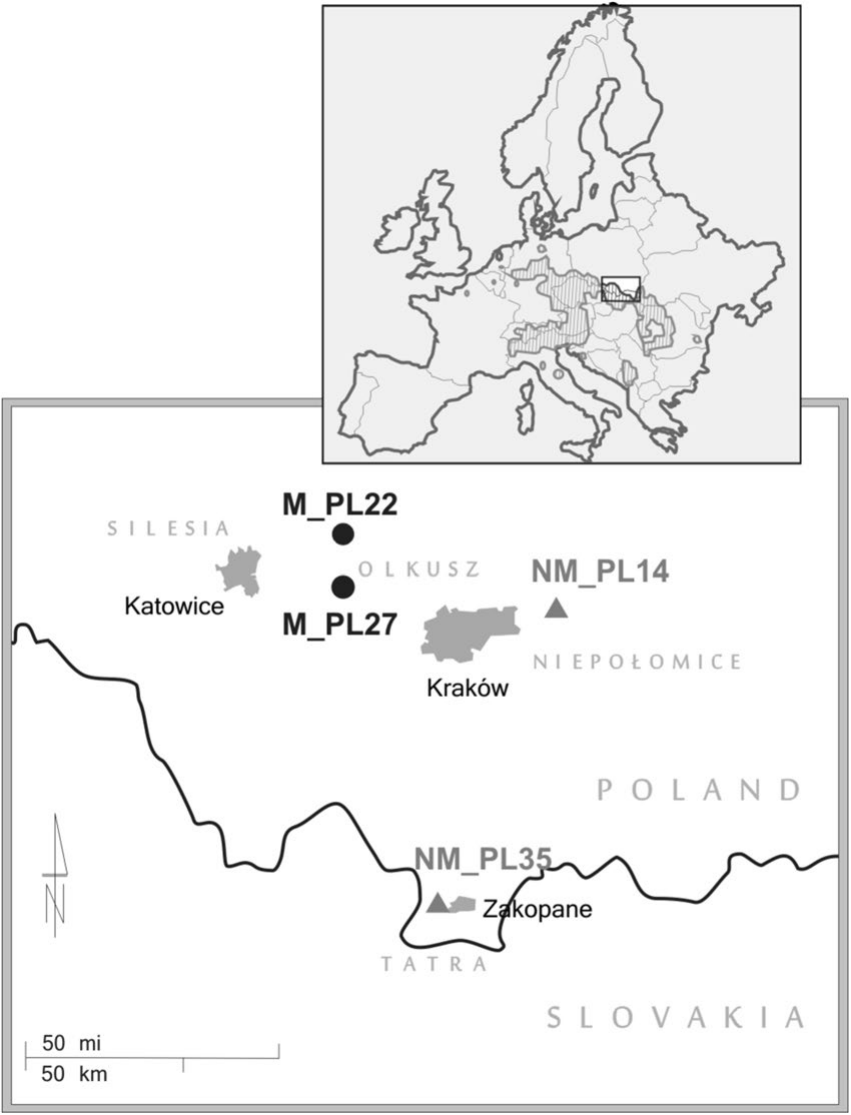
Locations of study sites of *Arabidopsis halleri*. The hatched area represents the distribution range of *A. halleri* in Europe (data from Atlas Florae Europaeae^79^). Black circles represent metalliferous (M), grey triangles non-metalliferous (NM) sites, grey shapes represent the area of indicated cities. For more details, see Table 1.

Here, we explore the genetic basis of plant adaptation to high soil concentrations of TMEs by comparing two metallicolous (from M habitats) and two non-metallicolous (from NM habitats) populations (Fig. 1 and Table 1) of the pseudometallophyte *Arabidopsis halleri* (L.) O’Kane and Al Shebaz. This outcrossing and also vegetatively reproducing Brassicaceae hypertolerates and hyperaccumulates Zn and cadmium (Cd)^27^. While hyperaccumulation of Zn in *A. halleri* is constitutive (i.e. species-wide), it appears to be population-specific for Cd^28,29^. Yet, a broad quantitative variation among populations has been observed for both traits under field and experimental conditions^20,28–30^. We used whole-genome re-sequencing of population pools (Pool-seq^31^) and mapped the obtained reads to a *de-novo* assembled draft reference genome of Swiss *A. halleri*. Next, we associated the allele frequencies of the identified single-nucleotide polymorphisms (SNPs) with environmental variables via environmental association analyses (EAA^2^). To complement this nucleotide-based approach and to account for a putatively polygenic nature of adaptation, we further used a gene-set enrichment analysis approach^32^. We present the SNPs, genes and pathways associated with different soil types and discuss our results in light of potential biological functions regarding (local) TME adaptation.

**Table 1:** The four sampled populations of *Arabidopsis halleri* with coordinates and average concentrations [ppm] of the five soil trace metal elements. See also Fig. 1.

Population	Location	Latitude [°N]	Longitude [°E]	Total Cd	Extractable K	Extractable Mg	Extractable Pb	Extractable Zn
M_PL22	Olkusz-Bukowno	50.282800	19.478717	23.7	87.3	425.0	668.5	1595.7
M_PL27	Olkusz-Galman	50.198367	19.538817	137.7	60.4	2445.0	2713.2	3889.4
NM_PL14	Niepołomice Forest	50.108833	20.367467	0.5	103.8	201.3	10.9	16.7
NM_PL35	Tatra Mountains	49.287056	19.879417	0.3	99.1	73.0	12.0	6.4

## Results

### *De novo* reference genome of *Arabidopsis halleri*

To map our reads to a reference genome that is phylogenetically closer to the sampled populations than the available assembly of a recently published Japanese accession^33,34^, we created a *de novo* assembly of two Swiss specimens. The Illumina sequencing of these two libraries led to 76*10^6^ paired-end and 113*10^6^ mate-pair reads. After quality trimming and filtering, 73% of the reads were used for the assembly process. Of those, 68% were incorporated in the final assembly. The draft reference genome (Ahalleri_CH_v2) established in this study was 164.6 Mb in size and therefore accounts for 66% of the estimated genome size of *A. halleri* (250–255 Mb)^33^. It consisted of 40 345 scaffolds, included ca. 25 kb Ns, N50 was 82 799 bp, and the largest scaffold 774 kb. Of the predicted genes, 26 249 were larger than 67 amino acids and 16 088 could be functionally annotated. In order to assess the completeness of the genome assembly, we ran BUSCO v2.0.1^35^, which revealed 1312 complete and single-copy (91.1%), 16 complete and duplicated (1.1%), 52 fragmented (3.6%) and 60 missing (4.2%) orthologs.

### Metalliferous sites differ in their history and TME concentrations

In order to cover the range of demographic clusters and the highly diverse ecological settings that characterise *A. halleri* populations, we selected two anthropogenic M locations at low altitude (M_PL22 and M_PL27), one NM sub-alpine location (NM_PL35), and the only known NM lowland location in the study area (NM_PL14, Fig. 1). *Arabidopsis halleri* populations from these four locations hypertolerate and hyperaccumulate TMEs^9,21^. However, the history and soil composition of the four sites differ. M_PL22 is located in the vicinity of the Bolesław Mine and Metallurgical Plant (still operational) on abandoned farmland^36^, whereas M_PL27 is located in the area of an open-cast Zn and Pb ore mine (closed in 1912), with mining dating back to the 14^th^ century (https://szukajwarchiwach.pl/search?q=galman%20XSKANro%3At&order).

**Figure 2:**
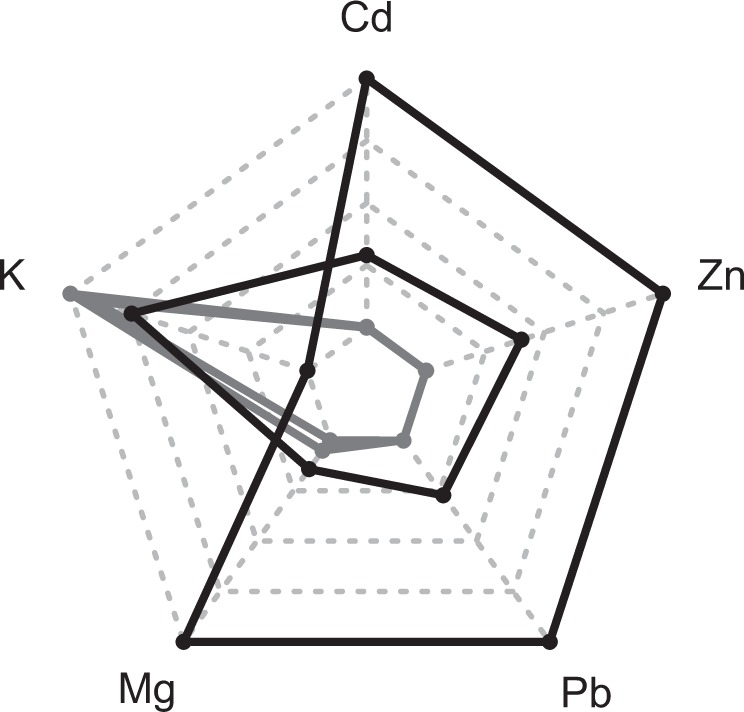
Relative soil concentrations of elements that differed between the investigated metalliferous (M, black) and non-metalliferous (NM, grey) sites of *Arabidopsis halleri*. Each of these elements had a significantly different concentration in both metalliferous sites compared to both non-metalliferous sites (one-way ANOVA, *P* < 0.001). The outer perimeter indicates the maximum and the central perimeter the minimum value per indicated element.

Since we were mainly interested in adaptation to different soils, we determined soil variables that significantly differentiated M and NM sites. In particular, we found that total Cd (F_3,11_ = 125, *P* = 4.7*10^−7^), extractable (surrogate for bioavailable) potassium (K) (F_3,11_ = 18.5, *P* = 5.8*10^−4^), extractable magnesium (Mg) (F_3,11_ = 331, *P* = 1.0*10^−8^), extractable lead (Pb) (F_3,11_ = 501, *P* = 1.9*10^−9^), and extractable Zn (F_3,11_ = 218, *P* = 5.2*10^−8^) content clearly differed between both site types (Fig. 2). Accordingly, these five soil variables were considered as the discriminators of M and NM soils in our study and thus included in specific EAAs. In particular, site M_PL27 showed considerably higher Cd, Pb, and Zn soil concentrations than the other M site (M_PL22), and very low levels of these elements were found at NM sites (Fig. 2). Concerning macronutrients, lower extractable K and higher extractable Mg at both M sites further indicated a distorted nutrient supply.

### Climatic and soil variables are orthogonal

**Figure 3:**
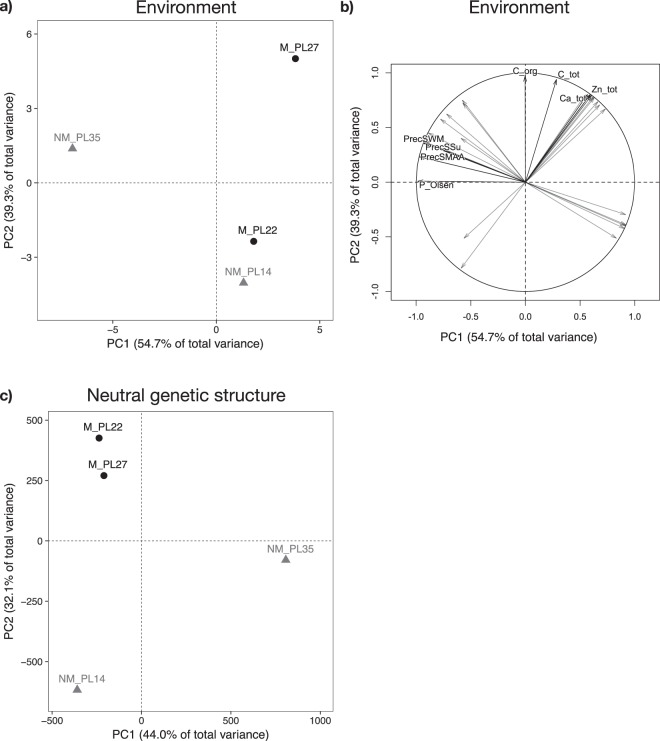
(**a**) Ordination plot of sampling locations generated by principal component analysis (PCA) of 31 environmental variables. (**b**) Environmental variables involved in the discrimination of the study locations defined by the first two principal components (PC). Only the top four contributing variables per PC are labelled, others are shown in grey. For more information see Supplementary Tables S1 and S11. (**c**) Neutral genetic population structure of the studied *Arabidopsis halleri* populations based on PCA. We used the allele frequencies of 500 000 randomly selected SNPs. (**a**,**c**) Black circles and grey triangles represent metalliferous (M) and non-metalliferous (NM) sites, respectively. (**b**) PrecSMAA – precipitation sum maximum annual amplitude; PrecSSu – precipitation sum summer; PrecSWM – precipitation sum wettest month; C_org – organic carbon content; C_tot – total carbon content; Ca_tot – total calcium content; P_Olsen – phosphorous content (Olsen method); Zn_tot – total zinc content.

To assess environmental differences between the four studied sites, we performed a principal component analysis (PCA) on climatic and soil variables. Metalliferous and NM locations were not clearly separated by their environmental conditions (Fig. 3a). However, the sub-alpine location (NM_PL35, lowest temperature and highest precipitation) and the Galman site (M_PL27, highest soil TME concentrations) were clearly distinct from the other two locations. With the exception of soil phosphorous (P) content, climatic variables dominated the contributions to PC1 (54.7% of total variance explained), while PC2 (39.3% of total variance explained) was mainly driven by soil variables (Fig. 3b, Supplementary Table S1).

### Next generation sequencing, mapping and SNP calling

From the four population pools, we obtained more than 10^9^ reads in total, 253*10^6^ reads per population on average. We could map 89% (coefficient of variation, CV = 1.5%) of reads to the reference genome, with an average (median) coverage of 155.1x (125x), 162.9x (131x), 122.8x (95x) and 165.3x (128x) for populations M_PL22, M_PL27, NM_PL14, NM_PL35, respectively (Supplementary Table S2). Each sequenced pool had >80% (average 83.9%, CV = 2.7%) of nucleotides covered at least 60x, that is each base is covered at least once per chromosome (30 diploid individuals per pool). In total, we found 3 724 036 raw SNPs. After stringent filtering we retained 2 529 878 high-confidence bi-allelic SNPs for downstream analyses.

### Neutral population structure does not reflect the environment

To identify the neutral genetic population structure, we performed a PCA with 500 000 random SNPs revealing a genetic distinction of metallicolous and non-metallicolous populations (Fig. 3c) along PC2 (32.1% of variance explained), but not along PC1 (44.0%). Furthermore, metallicolous populations were genetically more similar to each other than to non-metallicolous populations, and the two non-metallicolous populations were clearly separated (for genetic differentiation among populations see Table S3). Altogether, this indicates three genetic clusters: one formed by the two metallicolous populations and two represented by the two non-metallicolous populations. Importantly, the neutral genetic population structure did not mirror the environmental conditions, neither when only the five highly differentiated soil variables were used in the environmental PCA (Supplementary Fig. S1). Genome-wide population-specific Tajima’s *D* values were very similar and slightly positive for the four populations (median values NM_PL14 = 0.130, NM_PL35 = 0.123, M_PL22 = 0.123, M_PL27 = 0.145).

### Environmental association analyses reveal candidate genes involved in adaptation to soil TME concentrations

**Figure 4:**
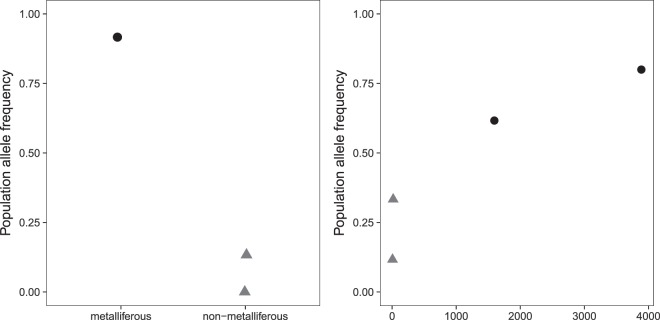
Allele frequency of a representative single nucleotide polymorphism (SNP) for **(a)** *MTPA2* in relation to the categorical variable Site-type, and (**b**) *SDP1* to the soil variable extractable zinc concentrations. Black – metallicolous population, grey – non-metallicolous population.

We used latent factor mixed models (LFMMs^37^) to identify the environment-driven genomic signatures of selection and found 12 927 SNPs (962 genes, Supplementary Table S4 and S5) to be associated with Site-type (M vs NM, example given in Fig. 4a). The SNP set identified as associated with Site-type in LFMM was strongly biased towards a *Z* statistic of 0.5 in Bayenv2^38^ (an alternative EAA method that we used for cross-validation of the LFMM results), the latter value indicating a strong correlation with the environmental variable. In contrast, a random set of 12 000 SNPs, in the majority representing putatively neutral sequence variants, yielded a rather uniform distribution of the *Z* statistic in Bayenv2 (Supplementary Fig. S2). This generally confirms the results of the LFMM analysis.

To identify SNPs associated with specific TMEs, we performed more detailed and targeted EAAs using the five soil variables that differed significantly between M and NM sites: total Cd, extractable K, Mg, Pb and Zn. We found 23 413 associated SNPs (1789 *A. thaliana* orthologous genes), 26 459 (1763), 23 460 (1685), 24 469 (1648) and 22 982 (1902, example given in Fig. 4b), respectively. Since we consider these five elements as the main discriminators of M and NM soils, the overlap of these genes (1375 genes, Supplementary Tables S6 and S7) may represent a genomic basis of adaption to M soils. The biological process “response to cadmium ion” was one of the most frequent gene ontolgy terms in these genes (Table S7).

### Three gene pathways enriched for a signature of adaptation to soil TME concentrations

To account for the putatively polygenic nature of adaptation, we tested whether certain gene sets were enriched for the *z* score, the test statistic of LFMM. In total, we found three pathways to be enriched: (1) solute carrier (SLC)-mediated transmembrane transport for Site-type, (2) translesion synthesis by POLH for all five soil variables, and (3) alpha-linolenic acid metabolism for all five soil variables (Supplementary Table S8).

**Figure 5:**
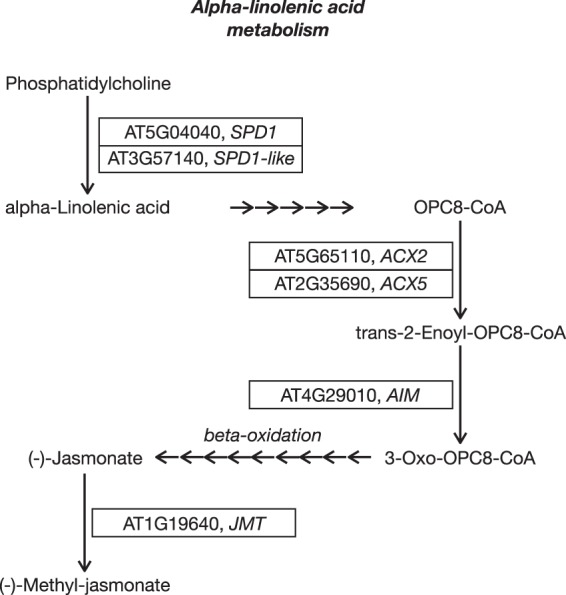
Simplified graphical representation of the alpha-linolenic acid metabolism (modified from Kyoto Encyclopedia of Genes and Genomes [KEGG] reference pathway). Boxes represent the identified candidate genes relevant to trace metal element adaptation in *Arabidopsis halleri*, names without boxes are substrates and products. Each arrow represents one enzymatic step.

Next, we marked all associated candidate genes that belong to one of the enriched pathways. This resulted in a total of 19 associated genes (hereafter referred to as ‘candidates’) that are the most likely involved in local adaptation to the soil characteristics in the study area (Table 2). In particular, we identified seven genes that belonged to the pathway enriched for Site-type, and 12 genes that were members of the two pathways enriched for the soil variables. One pathway is related to sequestration (solute carrier (SLC)-mediated transmembrane transport) while the other two are associated with response to stress, including OS (alpha-linolenic acid metabolism, Fig. 5), and DNA repair (translesion synthesis by POLH).

**Table 2:** Candidate genes identified as relevant for adaptation to metalliferous soil in *Arabidopsis halleri*.

Environmental variable	Gene^†^	Gene length [bp]	Number of SNPs in gene	Number of associated SNPs	Gene name	Enriched gene pathway	Tajima’s *D*^‡^			
							M_PL22	M_PL27	NM_PL14	NM_PL35
Site-type	AT1G18880	2351	35	2	Nitrate transporter 1.9/NFP2.9	SLC-mediated transmembrane transport	3.13	1.54	2.47	0.91
	AT1G47840	2800	78	2	Hexokinase 3	SLC-mediated transmembrane transport	0.89	0.46	1.61	3.14
	AT3G15380	3484	80	1	Choline transporter-like 1	SLC-mediated transmembrane transport	1.04	1.80	0.90	2.79
	AT3G23550	2385	61	2	Detoxification 18	SLC-mediated transmembrane transport	2.06	1.56	1.56	0.73
	**AT3G58810**	1140	5	4	Metal tolerance protein A2	SLC-mediated transmembrane transport	0.69	**−1.08**	1.53	0.10
	AT4G32510	3254	60	3	HCO3- transporter family	SLC-mediated transmembrane transport	2.39	2.15	0.69	3.03
	AT5G52050	1392	35	1	Detoxification efflux carrier 50	SLC-mediated transmembrane transport	1.27	0.66	1.07	2.10
Soil (Cd, K, Mg, Pb, Zn)	**AT1G19640**	2723	36	1, 1, 1, 1, 1	Jasmonic acid carboxyl methyltransferase	alpha-Linolenic acid metabolism	1.28	**−0.49**	1.57	0.27
	AT2G35690	3158	33	1, 1, 1, 1, 1	Acyl-CoA oxidase 5	alpha-Linolenic acid metabolism	1.03	1.61	1.83	3.12
	AT3G57140	2547	29	12, 9, 11, 12, 12	Sugar-dependent 1-like	alpha-Linolenic acid metabolism	2.16	0.36	2.34	0.30
	AT4G29010	5898	55	3, 3, 3, 3, 1	Enoyl-CoA hydratase/isomerase family	alpha-Linolenic acid metabolism	1.63	0.47	1.96	1.81
	AT5G04040	2649	22	2, 2, 1, 2, 2	Sugar-dependent 1	alpha-Linolenic acid metabolism	1.81	1.13	1.81	2.68
	AT5G65110	2654	33	3, 3, 3, 3, 3	Acyl-CoA oxidase 2	alpha-Linolenic acid metabolism	2.01	1.83	1.87	1.58
	**AT1G21690**	2618	100	1, 1, 1, 1, 1	Replication factor C 4	Translesion Synthesis by POLH	**−0.97**	**−0.92**	**−0.01**	1.83
	AT2G29070	1686	48	2, 3, 1, 2, 3	Ubiquitin fusion degradation UFD1 family protein	Translesion Synthesis by POLH	1.80	0.83	2.62	1.73
	AT3G02920	2293	20	8, 9, 8, 8, 7	Replication protein A, subunit RPA32	Translesion Synthesis by POLH	2.68	2.39	1.42	3.13
	AT3G53230	3358	45	2, 2, 2, 2, 1	ATPase, AAA-type, CDC48B protein	Translesion Synthesis by POLH	3.01	0.70	2.89	2.68
	AT5G03340	3319	30	1, 1, 1, 1, 1	ATPase, AAA-type, CDC48C protein	Translesion Synthesis by POLH	0.79	1.12	0.91	1.45
	AT5G27740	3162	114	3, 5, 3, 5, 5	Replication factor C 3	Translesion Synthesis by POLH	1.21	1.89	2.16	3.67

^†^Candidate genes with a negative Tajima’s *D* in at least one metallicolous population are marked in bold.

^‡^Negative Tajima’s *D* values are marked in bold.

All these genes contain SNPs that are associated with Site-type (metalliferous [M] vs non-metalliferous [NM]) or one of the five soil-specific variables and are members of an enriched gene pathway.

### Signatures of positive selection and few non-synonymous substitutions

Three candidates had a negative Tajima’s *D* in at least one metallicolous population, indicating departure from neutral expectations by recent positive selection (likely less than 10 000 generations ago^39^). The alternative explanation of a recent population expansion can rather be excluded as the overall population-specific Tajima’s *D* values were slightly positive while the observed gene-specific Tajima’s *D* values were in the lower quantile distribution (Table 2, Supplementary Fig. S3). In particular, *RFC4* (Replication Factor C4; AT1G21690) had a negative Tajima’s *D* in both metallicolous populations (M_PL22: −0.97, percentile = 0.0016; M_PL27: −0.92, percentile = 0.00008) and close to 0 or positive in non-metallicolous populations. *JMT* (jasmonic acid carboxyl methyltransferase; AT1G19640) and *MTPA2* (metal tolerance protein A2; AT3G58810) had negative Tajima’s *D* in the population originating from the most contaminated site M_PL27 (*JMT*: −0.49, percentile = 0.0005; *MTPA2*: −1.08, percentile = 0.00004).

To evaluate how the identified variants potentially affect the respective proteins, we assessed the predicted effects of the associated SNPs on the protein structure of the 19 genes. Most SNPs were either synonymous or intron variants. Only one of 15 Site-type associated candidate SNPs and nine of the 42 soil-associated SNPs were non-synonymous (NS) substitutions, respectively, corresponding to 20.7% of SNPs on average (Supplementary Tables S9, S10). In two of the three genes (*JMT* and *RFC4*) with a signature of positive selection (negative Tajima’s *D* value), a single SNP out of a total of 36 and 100 SNPs, respectively, generates an intron variant associated with all soil variables. *MTPA2* is the only identified transporter and had the lowest number of SNPs (five) identified, but four of these were associated with Site-type. One of these four SNPs is NS. We predicted the secondary structure of the MTPA2 protein to be an alpha helical transmembrane protein. The NS SNP associated with Site-type in *MTPA2* causes replacement of Isoleucine at position 138 by Leucine (I138L, change in the side chain conformation) in the first transmembrane helix.

## Discussion

In the present study, we searched for adaptive genetic changes that have evolved to allow the Brassicaceae *Arabidopsis halleri* to grow on M soils that were polluted with TMEs by mining since the late medieval times or more recently (<100 years ago) by industry^40^. Using four populations to detect the signature of local adaptation is at the lower end of statistical power. However, we think that this is counterbalanced by the categorical sampling design (replicated populations of M and NM sites) and the strong selection pressure that such TME-enriched soils exert on plants, complemented by the genome-wide perspective. Furthermore, the selected populations exhibit a wide range of TME concentrations in plant shoots and represent the genetic diversity and recently refined population genetic structure of *A. halleri* in southern Poland^21^.

By combining approaches targeting signatures of selection at the level of single nucleotides, genes and functional pathways, we identified 19 genes in three functional pathways (Table 2) that are likely to play roles in adaptation to TME-rich soils. This combination of approaches allowed us to avoid subjective biases^41^ and should have largely reduced the number of false positives. Given the possible multi- to polygenic nature of TME adaptation^11,23,28,29^ (but see^8^) potentially including a high number of candidate genes, this combination of approaches further allowed reducing the number of candidate genes for follow-up molecular studies. Most of the identified genes are members of transmembrane transport and stress signalling pathways, which indicates that both allocation and detoxification of TMEs are important physiological processes in adaptation to M soils.

Since we were interested in variants that increased or decreased in frequency as a result of positive selection, we here focus on three candidate genes: *MTPA2* (AT3G58810), *JMT* (AT1G19640), and *RFC4* (AT1G21690). Besides being associated to soil factors in EAAs and being members of gene pathways that are enriched for soil adaptation, these three candidates also have negative values of Tajima’s *D* in at least one metallicolous population. We consider these three genes to be our strongest candidates for metal stress adaptation. Below, we discuss them in the context of the enriched pathways and their physiological function.

### Metallicolous populations show alterations in ion transmembrane transport genes

The metal tolerance protein A2 (*MTPA2*/*MTP3*, AT3G58810) is involved in the transmembrane transport and vacuolar metal sequestration of divalent cations^42^. It is located in the vacuolar membrane (tonoplast), involved with Zn^2+^ homeostasis and confers tolerance to excess Zn^42^. This gene is a potential target of the Fe-deficiency induced transcription factor 1 (*FIT1*), a key regulator of Fe-deficiency responses^43^. However, there was no difference in the Fe soil content among our sites. Also, a recent study in *A. halleri* showed that *FIT1* expression is not altered under Zn treatment^28^. Thus, the FIT1 transcription factor seems not to be involved in adaptation to the M locations investigated herein.

It is remarkable that we found a signal of selection (negative Tajimas *D*) for *MTPA2* only in population M_PL27. Interestingly, M_PL27 was the most contaminated site in our study and had more than twice as much extractable Zn compared to the other M site M_PL22 (3900 *versus* 1600 ppm, respectively, Fig. 2). Furthermore, mining in the region of M_PL27 has been reported as early as the 14^th^ century. The name of the region, Galman, actually means ‘zinc ore’ and is likely derived from the centuries-long mining activity (https://szukajwarchiwach.pl/search?q=galman%20XSKANro%3At&order). Accordingly, the weaker and much younger (ca. 100 years^40^) selection pressure at M_PL22 seemed to be insufficient to create a strong selective sweep and thus resulted in a non-negative Tajima’s *D* in this population.

To date, no tertiary structure of the MTPA2 protein has been resolved, thus preventing positioning of the I138L substitution that we observed. However, using the Scratch protein structure prediction software, we could predict the secondary structure and found that the I138L substitution is located in a transmembrane helix. Furthermore, the alternative allele of the responsible NS SNP is at high frequency (>0.9) in both metallicolous populations and absent or at low frequency in both non-metallicolous populations (Fig. 4a, Supplementary Table S9). Our findings indicate that a modified version of this divalent cation transmembrane transporter is necessary for adaptation via presumed different sequestration of Zn^2+^, which is the main contaminant at the studied M sites. A similar role was recently suggested for another member of the *MTP* gene family, *AhMTP1*^30,44–46^. The well described *MTP1* (also known as *ZAT1* or *CDF1*) gene, encoding Zn^2+^ transporters involved in vacuolar sequestration, is considered a key component of hypertolerance to elevated Zn concentrations in *A. halleri*^24,47^. Still, several studies have demonstrated that different evolutionary fates, some of them not concurring with increased Zn tolerance, are likely to take place for the up to five paralogs of the *MTP1* in *A. halleri*^30,48,49^. Our study suggests that the related *MTPA2* has also played a role in the adaptive evolution of Zn tolerance in the species.

Another transporter that was identified as a candidate gene that is directly involved in ion homeostasis was choline transporter-like 1 (*CTL1*, AT3G15380). This gene regulates the expression pattern of different ion transporters through the modulation of vesicle trafficking^50^ and is closely associated with auxin signaling in the control of plant developmental processes^51^. Although *CTL1* was significantly associated with Site-type in our analysis, considering its positive Tajima’s *D* value it seems unlikely that this gene was under direct positive selection (Table 2).

### Stress signalling is putatively altered in response to high TME soil concentrations

Jasmonic acid (JA) and salicylic acid (SA) are two plant hormones that have widespread signalling roles with regard to many biotic and abiotic stresses which can cause oxidative damage^52^. In relation to oxidative stress, SA amplifies the oxidative signal and JA limits the oxidative lesion spreading^53,54^. Two candidate genes, *SDP1* and *SDP1-like* (AT5G04040 and AT3G57140), are located at the beginning of the enriched alpha-linolenic acid metabolism pathway that leads to the synthesis of JA and are responsible for generating the substrate alpha-linolenic acid (Fig. 5). It is striking that EAA detected six genes within this enriched gene pathway to be associated with the five soil-specific variables that best discriminate between M and NM sites. Accordingly and in agreement with other studies showing that elevated TME concentrations often result in oxidative stress^8–10^, we conclude that TME-induced oxidative stress is the most likely selective force. However, selection of altered JA responses can also be imposed by other stressors, including herbivory or drought. While climatic factors were not correlated with soil-related factors (Fig. 3b) and are therefore unlikely to be the selective force driving the identified patterns, potential effects of herbivory or pathogens at M sites cannot be excluded. Thus, this latter hypothesis needs further testing.

The second candidate that has a negative Tajima’s *D* in at least one metallicolous population, is jasmonic acid carboxyl methyltransferase (*JMT*, AT1G19640). Similar to *MTPA2*, its Tajima’s *D* was negative only for the population from medieval mining of natural Zn and Pb ore outcrops (M_PL27), suggesting that this is an ancient allele. On the one hand, the single associated SNP leading to an intron variant might not alter the protein or the protein’s expression. On the other hand, it is also possible that it creates a different splice variant or plays a role in the candidate’s regulation via different binding of transcription factors^55^. However, *JMT* has a direct connection to TME-induced stress. Methyl-JA has been shown to improve reactive oxygen species (ROS) scavenging through an enhanced antioxidant defence system^56^, linking it to OS response. It has also been shown to alleviate Cd-induced photosynthetic damage^57^, assigning a more prominent role to the molecule in plants growing on Cd-rich soils. Different to JA, however, methyl-JA does not control for lesion spread during cell death events triggered by OS^58^. Such lesions are a phenotypic hallmark of local TME adaptation and were shown to occur less often in adapted genotypes (from M sites in the Olkusz region) of another pseudometallophyte of the Brassicaceae family, *Biscutella laevigata*, compared to its non-tolerant genotypes (from NM habitats)^5^. Since *JMT1* and another five of the 19 candidates with associated SNPs are part of the alpha-linolenic acid metabolic pathway that ultimately leads to JA synthesis, we hypothesize that the levels of JA are balanced differently in adapted plants via methyl-JA and JA levels.

### DNA repair pathways show genetic variation associated with variation in soil TME concentrations

The third candidate, Replication factor C4 (*RFC4*, AT1G21690), is the only candidate with a negative Tajima’s *D* in both metallicolous populations. Despite this clear signal of selection, we identified only a single associated SNP that results in an intron variant (Supplementary Table S10). The protein RFC4 is part of the replication machinery and belongs to the ATPase family associated with various cellular activities (AAA)^59^. The other candidates that belong to the enriched ‘translesion synthesis by POLH’ pathway are involved in protein quality surveillance (*UFD1*, AT2G2970) or also in the replication machinery (e.g. *RPA32*, AT3G02920). Via translesion synthesis, POLH is specific to resolve pyrimidine dimers, which result from UV radiation^60^. Our current knowledge of POLH does not indicate any relation to high TME soil concentrations. However, the associated genes ensure protein quality and DNA repair and might relate to stress in general.

### Improved European *A. halleri* reference genome

As a backbone of our bioinformatic analyses, we established our own *de novo* assembled draft reference genome (Ahalleri_CH_v2) for mapping of the obtained reads. It represents a major improvement of the first *A. halleri* ssp. *halleri* draft genome used in Rellstab *et al*.^61^. Except for total assembly size, its assembly statistics are better than the first version of the assembly of the Japanese *A. halleri* ssp. *gemmifera* genome^62^, but inferior to its second version^33^, which was enhanced by adding long mate pair libraries of an inbred line. As Polish populations of *A. halleri* are genetically closer to Swiss than to Japanese populations^34^, and because the BUSCO analysis revealed a largely complete assembly (91.1%), we used the former as a reference.

### Future directions based on this study

While the list of potential candidate genes in our study was limited to known *A. thaliana* orthologs, we found candidate genes that presumably play a key role in adaptation to TME-rich soils. As some of these genes have not previously been linked with adaptation to heavy metals, we can only hypothesize about the mechanism and role that these genes might have in the identified pathways. *In silico* data mining can only provide a list of the most promising genes that have to be investigated in more detail. To clarify whether the identified genes are indeed important for adaptation to M soils, further functional proof is needed. This could for example be achieved using knock-out lines or reciprocal transplant experiments to show that metallicolous and non-metallicolous genotypes and their respective alleles actually have a fitness advantage in their home environment. Such a transplant experiment that also involves our study sites and populations is currently ongoing.

## Conclusion

Using whole-genome Pool-seq and environmental data, we found genomic signatures of adaptation to metalliferous soils by means of environmental association analyses. These associations were substantiated by identifying several gene networks, linked to transmembrane transport and response to stress, showing involvement in the local adaptation of *A. halleri* populations from southern Poland to high soil TME concentrations.

## Materials and Methods

### Reference genome of *Arabidopsis halleri*

To map our obtained sequences (see below) to a reference, we assembled and annotated our own draft reference genome of European *A. halleri*. For this, we used Illumina paired-end and 3 kb mate-pair libraries and plants from two Swiss populations (Aha11 and Aha18) described in Fischer *et al*.^63^. For details of the assembly process, see Supplementary Methods.

### Sampling and environmental data

Sampling included four locations of *A. halleri* in southern Poland (Fig. 1 and Table 1): two anthropogenic M locations in the Olkusz region (M_PL22 and M_PL27), one NM location in Niepołomice Forest (NM_PL14) – which is similar in climate to both M sites (Fig. 3a,b) – and one sub-alpine NM location in the northern foothills of the Tatra Mountains (NM_PL35). A recent study showed that plants from Olkusz and Niepołomice Forest regions belong to the same higher-level genetic cluster, while samples from the Tatra region were assigned to another cluster^21^. The latter cluster is considered to be the most ancestral and has the highest estimate of effective population size of all populations from southern Poland^21^. At each site, we collected leaves of 30 *A.halleri* plants every 4 m along transects to avoid potential clones and dried them on silicagel. We further collected three topsoil samples to a depth of 10 cm at each site using a cylinder of 7 cm diameter, followed by careful removal of the organic horizon.

To assess the soil and climatic environmental conditions at the study sites, we used (i) chemical data from local soil profiles and (ii) climatic factors (precipitation and temperature) from existing databases. For details on soil chemical analysis and climate data processing see Supplementary Methods. In total, we measured 16 soil variables (organic and total C, total N, available P, total and exchangeable Ca, Cd, K, Mg, Pb, and Zn) that are averages of three independent topsoil samples (Supplementary Table S11). We also generated 15 bioclimatic variables from the monthly temperature and precipitation data, averaged over the past 20 years (Supplementary Table S11).

To describe the environment, we performed a PCA with all 31 variables using the R package FactoMineR^64^ in R 3.3.4^65^. To identify soil variables that were significantly different between M and NM sites, we performed one-way analyses of variance (ANOVAs) on linear models of the form ‘soil variable ~ population’ with the R-package MASS^66^. We tested for normal distribution of the residuals using the Shapiro test and further tested for homoscedasticity using the Breusch-Pagan test as implemented in the R package olsrr^67^. If those model assumptions were violated, we Box-Cox transformed the corresponding soil variable prior to ANOVA. Finally, we repeated the above described PCA only with the soil variables that were significantly different between M and NM sites.

### Next-generation sequencing (NGS) and data processing

DNA extraction, library preparation, and next-generation sequencing followed the description in Supplementary Methods. We used a pooled sequencing approach (Pool-seq)^31^ that has been shown to deliver accurate allele frequencies in the studied species^61^.

We trimmed off adapter sequences of the Illumina Pool-seq data using cutadapt 1.9^68^. We then mapped the reads to our reference genome of *A. halleri* using Bwa-mem 0.7.12^69^ and Samtools 1.3^70^, and filtered the resulting BAM files for reads of mapping quality ≥10 with Bamtools 2.4.1^71^. After removing duplicate reads, adding read groups, and indexing the BAM files using Samtools and Bamaddrg (https://github.com/ekg/bamaddrg), we called genetic variants using Freebayes 1.0.1^72^. We used the pooled discrete mode for Pool-seq data, set ploidy to 60, the minimum alternate fraction to 0.083, minimum coverage to four, and maximum coverage to 10 000.

We filtered the resulting SNP list for bi-allelic loci using vcflib 1.0.1 (https://github.com/vcflib/vcflib) and for quality (PHRED scale) >30, minimum mapping quality >40, read depth <1200, individual sample depth >60, quality/read depth >0.25, no missing data and minimum (4) and maximum (236) alternate allele counts using bcftools^73^ (https://github.com/samtools/bcftools). Additionally, we removed all non-variant sites using a custom Python3 script. The complete list of options and parameters can be found in Supplementary Table S12.

### Neutral population structure and environmental association analyses

Information on neutral genetic population structure, in particular how many genetic clusters (*K*) comprised our sample, was required to appropriately parameterize the subsequent EAA. To infer the neutral population structure, we randomly chose 500 000 SNPs from our data set and used their allele frequencies to perform a PCA with FactoMineR.

Using LFMM^37^ incorporated in the R package Lea2^74^, we performed 12 runs (5000 burn-in cycles and 10 000 additional cycles) per environmental variable for *K* = 2–4 to account for neutral population structure. We used the categorical variable ‘Site-type’ (coded as 1 or 0), which denotes M or NM sites, respectively, and the soil variables (described above) as environmental variables. Following Lea2 instructions, we combined the results of the 12 runs using the median *z* score and calculated the genomic inflation factor (λ) for all *K*s. For all six variables, the lowest λ was at *K* = 3, which is in concordance with the neutral genetic population structure (see Results). Therefore, we used the LFMM results for *K* = 3 and corrected the *P* values with the corresponding λ value. We set the false discovery rate to 1%.

We cross-evaluated the LFMM results using Bayenv2^38^ in pooled mode. To construct the variance–covariance matrix, we randomly sampled 20% of all SNPs and saw the matrix converging after 32 000 iterations. Subsequently, we performed three runs with different random seeds for the set of SNPs associated with Site-type in LFMM. Additionally, we randomly selected 12 000 SNPs, excluding SNPs that were significantly associated in LFMM, and performed three runs as a random control. This number of random SNPs was chosen to match the SNPs found to be associated in LFMM analyses (see Results). We then assessed the overlap of the *Z* statistic histograms of the SNPs associated in LFMM and random sets to estimate the performance of the LFMM analysis.

In order to be able to functionally interpret our gene-based analyses, we focused on functionally annotated *A. thaliana* orthologs. Using “intersect” of Bedtools and Unix’ “awk” commands, we added the *A. thaliana* orthologue gene name to each SNP. Each annotated gene that contained at least one environmentally associated SNP was considered a candidate gene for adaptation to variation in soil TME concentrations.

### Gene set enrichment analyses

To identify signatures of putatively polygenic adaptation, we used the Polysel pipeline for gene set enrichment analysis^32^. We obtained GeneIDs, BSID and the BSID to GeneID lists for *A. thaliana* from the repository at https://www.ncbi.nlm.nih.gov/biosystems/. We based our Polysel analyses on the maximum absolute LFMM *z* scores per gene (using all SNPs, not only the significantly associated). We added the NCBI Biosystems GeneID using a custom Python3 script. We applied the default settings of the pipeline and a post-pruning *Q* value cutoff of 10%. As we were primarily interested in adaptation to edaphic conditions, we performed this analysis for (i) Site-type and (ii) the five soil variables that significantly differed between M and NM sites.

### Population genomic analyses, gene diversity and SNP annotation

We used custom python3 scripts to calculate nucleotide diversity *π* and Watterson’s *θ*_W_^75^ to estimate Tajima’s *D*^76^ for every gene in every population. A negative Tajima’s *D* value indicates an excess of rare alleles and is usually interpreted as either a signature of a selective sweep or a recent population expansion, e.g. after a bottleneck.

To infer the possible effect and impact of a single substitution, we used SnpEff^77^. We added *A. halleri* to the SnpEff database following the developer’s instructions and annotated our final, stringently filtered vcf file. We used this annotated vcf file with our candidate bed files to extract the ‘Effect’ (e.g. intron-variant, non-synonymous (NS) variant) and ‘Impact’ (e.g. moderate, high) from the SnpEff annotation for the LFMM-derived SNPs. To identify whether a NS SNP has an influence on protein structure, we ran the online tool ‘Scratch Protein Predictor’ and used the SSpro (3 Class) and SSpro8 (8 Class) secondary structure predictor therein^78^.

## Electronic supplementary material

Supplementary methods and figures

Supplementary Table S1

Supplementary Table S2

Supplementary Table S3

Supplementary Table S4

Supplementary Table S5

Supplementary Table S6

Supplementary Table S7

Supplementary Table S8

Supplementary Table S9

Supplementary Table S10

Supplementary Table S11

Supplementary Table S12

## Data Availability

The reference genome assembly is available at NCBI GenBank under PRJNA492199. Raw reads used for the reference genome assembly are available at NCBI SRA under SAMN10130990 (Aha18_mp and Aha11_10B combined), SAMN10095121/SRR8083399 (Aha18_mp), and SAMN10095118/SRR8083441 (Aha11_10B). Raw reads for SNP calling of the four population pools are accessible at NCBI SRA under projectID PRJNA495924, with reads for PL14 under SAMN10234903/SRR0840827, for PL22 under SAMN10234904/SRR8040826, for PL27 under SAMN10234905/SRR8040825, and for PL35 under SAMN10234906/SRR8040824).
