# Supplementary material for: Transmembrane transport and stress response genes play an important role in adaptation of Arabidopsis halleri to metalliferous soils
Source: Sci Rep. 2018 Oct 31;8:16085. doi: 10.1038/s41598-018-33938-2 (PMC6208402; doi:10.1038/s41598-018-33938-2)
Supplement: Supplementary file 1 — Supplementary methods and figures [file 41598_2018_33938_MOESM1_ESM.pdf]

Transmembrane transport and stress response genes play an important role in adaptation of *Arabidopsis halleri* to metalliferous soils

Christian Sailer[1,2*], Alicja Babst-Kostecka[3], Martin C. Fischer[4], Stefan Zoller[5], Alex Widmer[4], Pierre Vollenweider[1], Felix Gugerli[1], Christian Rellstab[1]

[1] WSL Swiss Federal Research Institute, Birmensdorf, 8903, Switzerland
[2] Current address: John Innes Centre, Cell and Developmental Biology, Norwich, NR4 7UH, UK
[3] W. Szafer Institute of Botany, Polish Academy of Sciences, Krakow, 31512, Poland
[4] ETH Zürich, Institute of Integrative Biology, Zürich, 8092, Switzerland
[5] ETH Zürich, Genetic Diversity Centre, Zürich, 8092, Switzerland
* Christian.Sailer@jic.ac.uk, Tel. +441603 450 926

Supplementary Methods

Reference genome of *Arabidopsis halleri*

First, paired-end sequencing (2x 100 bp, insertion size 250 bp) was performed using a single natural individual of population Aha11 that was cultivated in the greenhouse (Aha11_10B) and showed low genetic diversity[1], which facilitates genome assembly. Second, we sequenced a mate-pair library (2x 50 bp, planned insert size 3000 bp, mean processed insert size 3689 bp) constructed from one individual grown from a single seed of population Aha18. Individuals of this population provided high quality DNA from leaves, which is important for the production of mate-pair libraries. Leaves were dried on silicagel and DNA was extracted with the DNeasy Plant Kit (Qiagen, Hilden, Germany). DNA quality was assessed using 1.5% agarose gels stained with GelRed (Biotium, Hayward, USA) and a Nanodrop 8000 (Thermo Fisher Scientific, Waltham, USA). DNA quantity was determined with a Qubit fluorometer (dsDNA BR, Invitrogen, Carlsbad, USA). The paired-end library was prepared and sequenced by the Quantitative Genomics Facility (D-BSSE, ETH Zürich/Basel, Switzerland) using 1/6 of a HiSeq2000 run. The mate-pair library was prepared and sequenced by GATC Biotech (Constance, Germany) using 1/3 of an Illumina HiSeq2000 (Illumina, San Diego, USA) run.

We used CUTADAPT[2] to trim forward and reverse raw reads for tags and adaptors. Then, Phred-type quality scores of Q20 were used for quality trimming with the FASTX toolkit (http://hannonlab.cshl.edu/fastx_toolkit). The separately trimmed forward and reverse reads were then re-synchronized to pairs with an in-house PERL script. Only paired sequences were used for further analysis. The quality-trimmed paired-end and mate-pair reads were subsequently assembled together with VELVET 1.2.08[3] with a kmer size of 41. Thereafter, the assembly was polished using SSPACE[4] for scaffolding and GAPFILLER v1.9[5] to fill gaps in scaffolds. Scaffolds with lengths below 200 bp were discarded.

Automated gene prediction and structural annotations were generated with the pipeline MAKER2[6] using the gene prediction tools SNAP[7], AUGUSTUS[8], and GENEMARK-ES[9]. All proteins from the *Arabidopsis thaliana* reference genome (TAIR10[10]) were used as protein homology evidence. All EST (expressed sequence tag) sequences for *A. thaliana* available on NCBI (https://www.ncbi.nlm.nih.gov/) were downloaded and included as alternative EST evidence. The model organism for repeat masking was set to *A. thaliana*. Two iterative MAKER2 runs were made to produce a final set of gene predictions and protein translations. Prior to the second iteration, a subset of the predicted genes from the first iteration was used to train and test the AUGUSTUS gene prediction model.

For functional annotation, the downloaded proteins of *A. thaliana* and the translated protein sequences resulting from the MAKER2 annotations were used as input for finding orthologous sequences with the tool OMA[11]. Only orthologues with a one-to-one relationship with *A. thaliana* were selected, one-to-many and many-to-many relationships were discarded. The genes with one-to-one orthologues received the functional annotation of the corresponding orthologue in *A. thaliana*.

In order to assess the completeness of the assembly we ran BUSCO v2.0.1[12] with the embryophyta database (containing 1440 BUSCO groups) downloaded from the BUSCO website. The Augustus optimization mode was activated with the species set to "arabidopsis".

DNA extraction, library preparation, and next-generation sequencing

We used 15 mg of silicalgel-dried leaf tissue for DNA extraction with a KingFisher Flex 96 (Thermo Fisher Scientific) and the sbeadex maxi plant kit (LGC

Genomics, Berlin, Germany). We checked the quality of the DNA on 1.5% agarose gels stained with GelRed and a Nanodrop 1000 (Thermo Fisher Scientific). We then quantified the DNA with a Quantus fluorometer (Promega, Madison, USA) and created four population pools consisting of equal amounts of DNA from 30 individuals, except for population NM_PL35, for which 29 extractions were available only. Library preparation with the KAPA HyperPrep Kit (Roche, Basel, Switzerland) and NGS (2x150 bp paired-end reads on two lanes of an Illumina HiSeq 2500) was performed by the Quantitative Genomics Facility (D-BSSE, ETH Basel, Switzerland).

Soil chemical analysis and climate data processing

Soil samples were air-dried and sieved with a 2 mm mesh. Organic carbon was assessed using a dry combustion technique with a LECO RC-612 (LECO, St. Joseph, USA). Total nitrogen was determined using the Kjeldahl method[13]; soil was digested in H_2SO_4 with Kjeltabs (K_2SO_4 + $CuSO_4$•$5H_2O$; Tecator Digestor Auto, FOSS, Hilleroed, Denmark) followed by distillation on a FOSS Tecator Kjeltec 2300 Analyzer Unit. The available phosphorus (Olsen phosphorous[14]) was measured with an ion chromatograph (Dionex ICS-1100, Thermo Fisher Scientific) following soil extraction with 0.5 M $NaHCO_3$. Total and exchangeable Ca, Cd, K, Mg, Pb, and Zn were extracted by digestion of ground soil in hot concentrated $HClO_4$ (FOSS Tecator Digestor Auto) or vortexing soil solution with EDTA[15], respectively. The concentrations of the extracted elements were determined using atomic absorption spectrometry (Varian AA280FS, Agilent Technologies, Santa Clara, USA) and the results were ascertained using certified reference soil material CRM048-050 and BCR-483 (Sigma-Aldrich). The recovery values (validation of method) ranged between 92 and 102% (n = 3).

Monthly data of precipitation and air temperature for the period 1997-2016 were obtained from the meteorological stations nearest to the four study sites: Maczki, Olewin, Kościelisko-Kiry, and Igołomia meteorological stations for M_PL22, M_PL27, NM_PL35, and NM_PL14, respectively. All stations were located within 15 km distance from the sites and the meteorological databases were obtained from The Polish Institute of Meteorology and Water Management – National Research Institute (IMGW-PIB).

Supplementary Figures

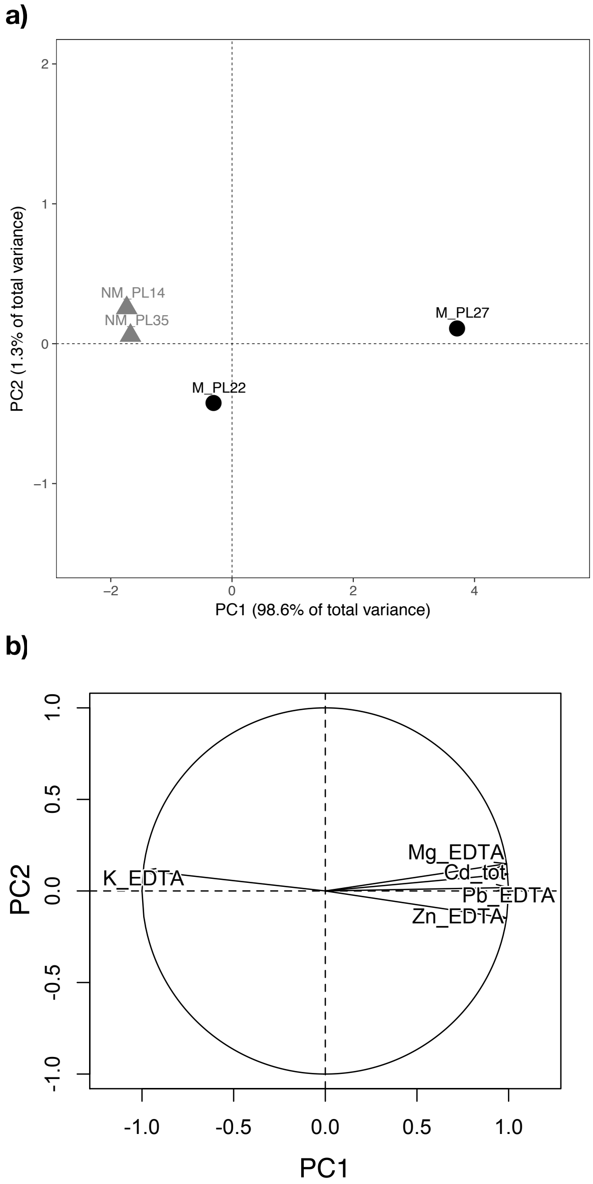

Supplementary Figure S1 a) Ordination plot of sampling locations of *Arabidopsis halleri* generated by a principal component analysis (PCA) using the five elements that significantly differed between metalliferous (M, black) and non-metalliferous (NM, grey) sites. **b)** Variables driving the discrimination along PC1 and PC2.

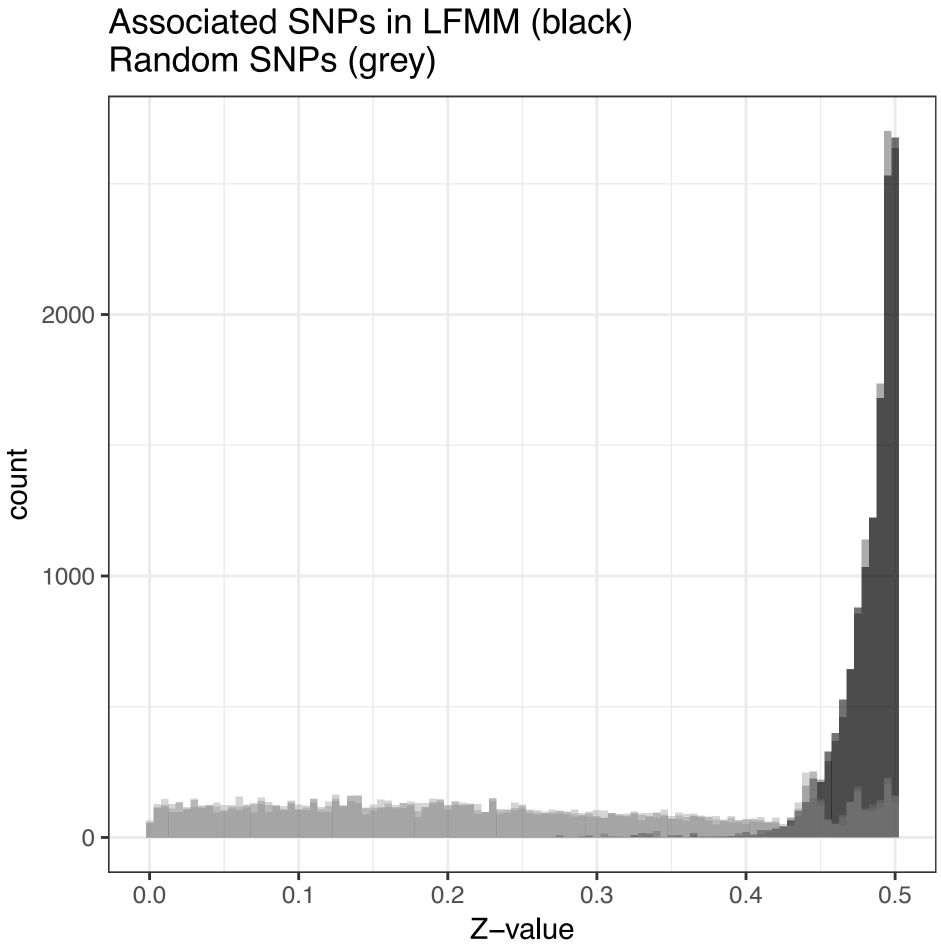

Supplementary Figure S2 Cross evaluation of LFMM[16] SNPs (associated to Site-type) with BAYENV2[17] in *Arabidopsis halleri*. Black bars represent three independent BAYENV2 runs with only the SNPs associated in LFMM, whereas grey bars represent three independent BAYENV2 runs with a random set of 12 000 SNPs. Note the clear black peak at the Z value of 0.5, indicating correlation with the environmental variable.

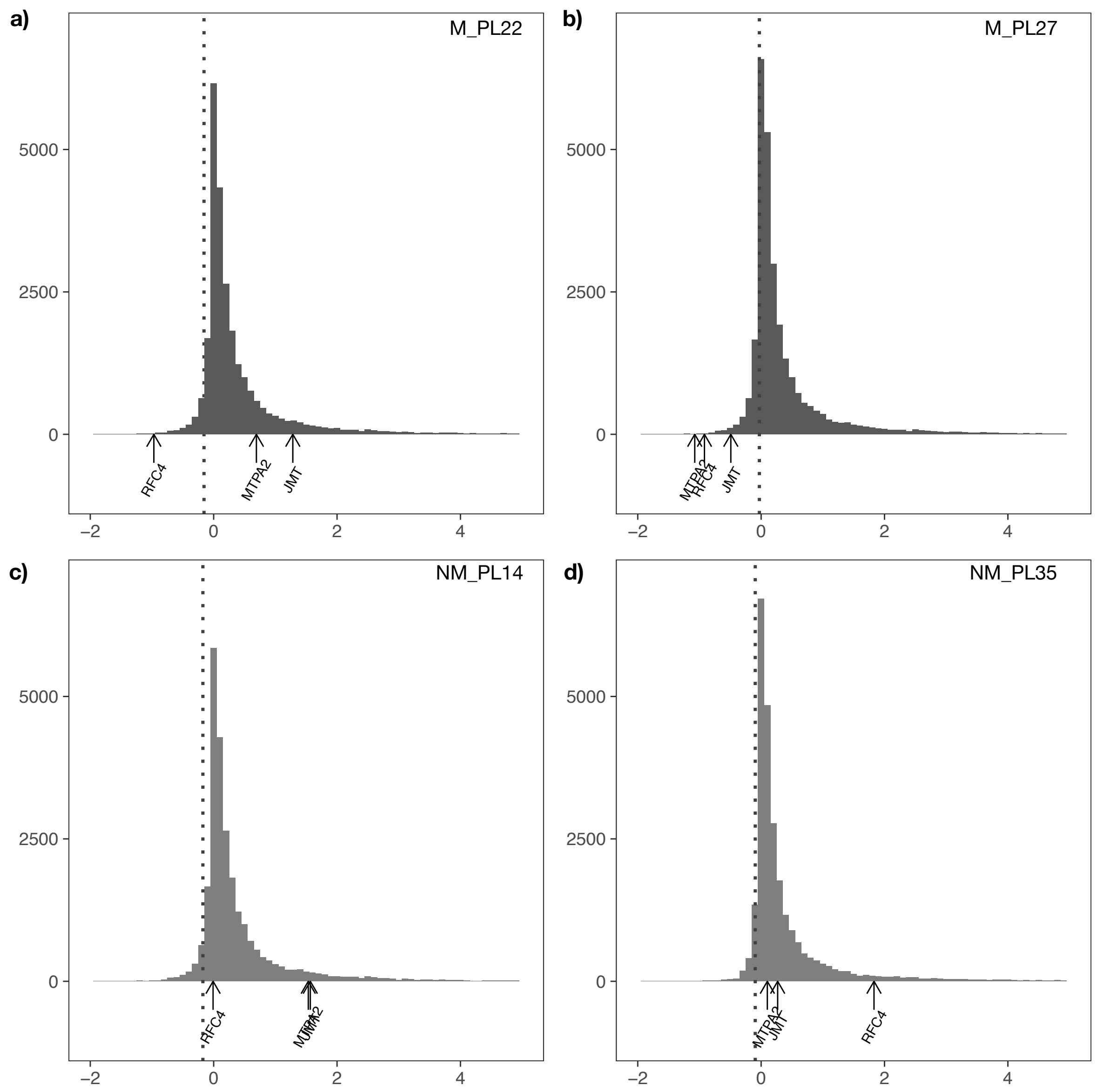

Supplementary Figure S3 Distribution of Tajima's *D* per gene across the genome (25 215 genes) of each population of *Arabidopsis halleri*. The dotted line indicates the 5% quantile. We consider negative Tajima's *D* values as likely due to positive selection (see main text). The values of the three candidate genes with a negative value in at least one population are indicated by arrows. Light grey – non-metallicolous populations: dark grey – metallicolous populations.

Supplementary Tables

List of supplementary tables. They can be downloaded as separate files.

Supplementary Table S1 Variable contribution to principle component (PC) 1 and PC2 of the principle component analysis (PCA) of environmental variables describing the sampling locations of *Arabidopsis halleri*. The table is sorted by the variable contribution to PC1 and the rank for PC2 is shown in the last column.

Supplementary Table S2 Summary of read mapping and depth of coverage (DOC). We assessed DOC after removing unmapped and duplicated reads from the Pool-seq data of *Arabidopsis halleri*. Mapping efficiency = mapped reads / total reads.

Supplementary Table S3 Pairwise F_{ST} values for all six *Arabidopsis halleri* population pairs.

Supplementary Table S4 Genes containing single nucleotide polymorphisms (SNPs) associated with the environmental variable Site-type. ‘Contig’ refers to the contig/scaffold of our *Arabidopsis halleri* reference genome including the start and end position of the gene. ‘Gene’ shows the *A. thaliana* orthologue TAIR ID.

Supplementary Table S5 Twenty most frequent gene ontology (GO) terms (biological processes only) of the genes identified in the environmental association analysis (EAA) using the environmental variable Site-type. See Table S4 for the complete gene list.

Supplementary Table S6 Genes containing single nucleotide polymorphisms (SNPs) associated with soil-specific (Cd, Mg, K, Pb, Zn content) environmental variables. ‘Contig’ refers to the contig/scaffold of our *Arabidopsis halleri* reference genome including the start and end position the gene. ‘Gene’ shows the *A. thaliana* orthologue TAIR ID.

Supplementary Table S7 Twenty most frequent gene ontology (GO) terms (biological processes only) of the genes identified in the environmental association analyses (EAA) using the soil-specific (Cd, Mg, K, Pb, Zn concentrations) environmental variables. See Table S6 for the complete gene list.

Supplementary Table S8 Summary of gene set enrichment analyses. One analyses per variable was run. ‘Setsize’ indicates the number of genes within each set/pathway, ‘setP’ is the P value for this set, ‘setQ’ is the false discovery rate (FDR) controlled Q value for this set, ‘setName’ refers to the pathway name.

Supplementary Table S9 Site-type associated SNPs of the candidates. Gene – TAIR ID, Ref – reference base, Alt – alternative base (SNP), Effect –likely effect this SNP causes, Impact –putative impact of that SNP, AF – population allele frequency of the alternative allele, M – metallicolous, NM – non-metallicolous, z-score – LFMM *z* score of that SNP, Contig, Start and End – Position of gene in Swiss *Arabidopsis halleri* reference genome.

Supplementary Table S10 Soil (Cd, Mg, K, Pb, Zn content)-associated single nucleotide polymorphisms (SNPs) of the candidates. Gene – TAIR ID, Ref – reference base, Alt – alternative base (SNP), Effect – likely effect this SNP causes, Impact – putative impact of that SNP, AF – population allele frequency of the alternative allele, M – metallicolous, NM – non-metallicolous, z-score – LFMM *z* score of that SNP, EA_ – environment associated SNP for indicated element, Contig, Start and End – Position of gene in Swiss *Arabidopsis halleri* reference genome.

Supplementary Table S11 Environmental variables used to describe the sampling locations of *Arabidopsis halleri*. We calculated and used variables of three categories and two classes: Temperature, Precipitation (Class 'Climate') and Soil chemical composition (Class 'Soil'). For the latter, total amount (tot), organic (org), and extractable fractions (EDTA) are presented. For class 'Climate' we show the abbreviations used in the main text.

Supplementary Table S12 Next Generation Sequencing data processing steps with set parameters. Script refers to the script name that we used. Options lists all options we set beside the default or which are changed default parameters.

References

1. Fischer, M. C. *et al.* Estimating genomic diversity and population differentiation – an empirical comparison of microsatellite and SNP variation in *Arabidopsis halleri*. *BMC Genomics* **18,** 69 (2017).
2. Martin, M. Cutadapt removes adapter sequences from high-throughput sequencing reads. *EMBnet.journal* **17,** pp. 10–12 (2011).
3. Zerbino, D. R. & Birney, E. Velvet: algorithms for de novo short read assembly using de Bruijn graphs. *Genome Res.* **18,** 821–829 (2008).
4. Boetzer, M., Henkel, C. V., Jansen, H. J., Butler, D. & Pirovano, W. Scaffolding pre-assembled contigs using SSPACE. *Bioinformatics* **27,** 578–579 (2011).
5. Boetzer, M. & Pirovano, W. Toward almost closed genomes with GapFiller. *Genome Biol.* **13,** R56 (2012).
6. Holt, C. & Yandell, M. MAKER2: an annotation pipeline and genome-database management tool for second-generation genome projects. *BMC Bioinformatics* **12,** 491 (2011).
7. Korf, I. Gene finding in novel genomes. *BMC Bioinformatics* **5,** 59 (2004).
8. Stanke, M. & Waack, S. Gene prediction with a hidden Markov model and a new intron submodel. *Bioinformatics* **19,** ii215–ii225 (2003).
9. Lomsadze, A., Ter-Hovhannisyan, V., Chernoff, Y. O. & Borodovsky, M. Gene identification in novel eukaryotic genomes by self-training algorithm. *Nucleic Acids Res.* **33,** 6494–6506 (2005).
10. Lamesch, P. *et al.* The *Arabidopsis* Information Resource (TAIR): improved gene annotation and new tools. *Nucleic Acids Res.* **40,** D1202–1210 (2012).

11. Altenhoff, A. M. *et al.* The OMA orthology database in 2015: function predictions, better plant support, synteny view and other improvements. *Nucleic Acids Res.* **43,** D240–249 (2015).
12. Simão, F. A., Waterhouse, R. M., Ioannidis, P., Kriventseva, E. V. & Zdobnov, E. M. BUSCO: assessing genome assembly and annotation completeness with single-copy orthologs. *Bioinformatics* **31,** 3210–3212 (2015).
13. Sáez-Plaza, P., Michałowski, T., Navas, M. J., Asuero, A. G. & Wybraniec, S. An overview of the Kjeldahl method of nitrogen determination. Part I. Early history, chemistry of the procedure, and titrimetric finish. *CRC Cr. Rev. Anal. Chem.* **43,** 178–223 (2013).
14. Olsen, S. R., Cole, C. V., Watanabe, F. S. & Dean, L. A. Estimation of available phosphorus in soils by extraction with sodium bicarbonate. *U.S. Department of Agriculture* 1–22 (1954).
15. Stefanowicz, A. M., Stanek, M., Woch, M. W. & Kapusta, P. The accumulation of elements in plants growing spontaneously on small heaps left by the historical Zn-Pb ore mining. *Environ. Sci. Pollut. Res. In.t* **23,** 6524–6534 (2016).
16. Frichot, E., Schoville, S. D., Bouchard, G. & François, O. Testing for associations between loci and environmental gradients using latent factor mixed models. *Mol. Biol. Evol.* **30,** 1687–1699 (2013).
17. Günther, T. & Coop, G. Robust identification of local adaptation from allele frequencies. *Genetics* **195,** 205–220 (2013).
